# Standard Hypercaloric, Hyperproteic vs. Leucine-Enriched Oral Supplements in Patients with Cancer-Induced Sarcopenia, a Randomized Clinical Trial

**DOI:** 10.3390/nu15122726

**Published:** 2023-06-12

**Authors:** Aura D. Herrera-Martínez, Soraya León Idougourram, Concepción Muñoz Jiménez, Rosa Rodríguez-Alonso, Rosario Alonso Echague, Sonia Chica Palomino, Ana Sanz Sanz, Gregorio Manzano García, María Ángeles Gálvez Moreno, Alfonso Calañas Continente, María José Molina Puertas

**Affiliations:** 1 Maimonides Institute for Biomedical Research of Cordoba (IMIBIC), 14004 Cordoba, Spain; sorayaleon3@hotmail.com (S.L.I.); carri2976@gmail.com (C.M.J.); rosarodriguezalonso@gmail.com (R.R.-A.);; 2 Endocrinology and Nutrition Service, Reina Sofia University Hospital, 14004 Cordoba, Spain; 3 Medical Oncology Service, Reina Sofia University Hospital, 14004 Cordoba, Spain; 4 General Surgery Service, Reina Sofia University Hospital, 14004 Cordoba, Spain

**Keywords:** oral supplements, leucine, cancer, malnutrition, sarcopenia

## Abstract

(1) Background: Malnutrition frequently affects patients with cancer, and it negatively impacts treatment tolerance, clinical outcomes and survival. Thus, appropriate nutritional screening and early nutrition support are extremely recommended. Currently, a significant number of oral supplements (OS) are commercially available; despite this, there is a lack of evidence for recommending specific OS, including leucine-enriched OS, for nutritional support in patients with cancer. (2) Aim: To compare the clinical evolution of patients with cancer (undergoing systemic treatment) that received standard hypercaloric, whey protein-based hyperproteic oral supplements vs. hypercaloric, hyperproteic leucine-enriched OS using a novel morphofunctional nutritional evaluation. (3) Patients and methods: This paper details an open-label, controlled clinical study in which patients were randomly assigned to receive nutritional treatment with whey protein-based hyperproteic oral supplements (control group) vs. hypercaloric, hyperproteic leucine-enriched OS (intervention group) during a twelve-week period. Forty-six patients were included; epidemiological, clinical, anthropometric, ultrasound (muscle echography of the rectus femoris muscle of the quadriceps and abdominal adipose tissue) and biochemical evaluation were performed. All patients received additional supplementation with vitamin D. (4) Results: Nutritional parameters (including bioimpedance, anthropometric, ultrasound and biochemical variables) of all included patients remained stable after the nutritional intervention. Extracellular mass tended to increase in the patients that received the leucine-enriched formula. Functionality (evaluated through the stand-up test) improved in both groups (*p* < 0.001). Prealbumin, transferrin levels and superficial adipose tissue increased in the control group (*p* < 0.05), while self-reported quality of life improved in all the evaluated patients (*p* < 0.001). (5) Conclusions: Nutritional support with hypercaloric, hyperproteic (with whey protein) OS and vitamin D supplementation were associated with the maintenance of body composition and improvements in functionality and in quality of life in the patients with cancer undergoing systemic treatment. No significant benefits were observed when a leucine-enriched formula was used.

## 1. Introduction

Cancer is one of the main causes of morbidity and the second cause of mortality worldwide. It is estimated that in 2040, there will be 28.4 million cancer cases globally. According to a recent report, 19.3 million new cancer cases were reported in 2020 worldwide; among them, 18.1 million excluded non-melanoma skin cancer, and around 10.0 million cancer deaths were reported [1,2]. According to the World Health Organization, one in five men and one in six women worldwide develop cancer during their life, and one in eight men and one in eleven women die from the disease [3].

Malnutrition and sarcopenia are frequent in patients with cancer; it occurs in 30–90% of patients, especially if it affects the gastrointestinal tract [4]. This is a multifactorial condition that includes inadequate/insufficient food intake, decreased physical activity, involvement of the gastrointestinal tract (local effects) by the tumor, psychological effects, the tumor per se, metabolic disorders, therapy-related adverse effects, alterations in nutrient metabolism and resting energy expenditure [5].

Sarcopenia implies fatigue and decreased muscle strength due to reduced skeletal muscle mass, which is accompanied by muscle atrophy and a decreased quality of muscle tissue. Specifically, the muscle fibers are replaced by fibrotic tissue, which results in increased fragility and impaired muscle function [3]. In this context, cancer is possibly the disease that most significantly favors muscle atrophy, resulting in the loss of functionality, especially in the elderly [4].

Recent studies suggested that tumor-derived inflammation and the resulting systemic inflammation are closely related to sarcopenia and cancer cachexia. Specifically, tumor cells release cytokines, inflammatory mediators and activated immune cells [6], resulting in systemic inflammation, muscle wasting and fat depletion [7]. This cancer-related inflammation originates an imbalance between protein synthesis and degradation, favoring the latter, and results in muscle loss [5].

Currently, there is no standardized treatment protocol for sarcopenia and cancer cachexia in patients undergoing systemic treatment. In most patients, it is necessary to increase energy intake; this increase alone does not correct sarcopenia and cancer cachexia due to several reasons including the inflammatory status of the patient. In this context, and when the digestive tract is functioning, the use of oral supplements (OS) is necessary. Currently, several formulas are available, but the evidence favoring one over the other is still lacking; in this context, specific strategies for nutritional supplementation should be explored, including leucine. This is a branched-chain amino acid (BCAA) that exerts downstream effects on muscle protein synthesis and promotes muscle protein anabolism [4].

The European Society for Parenteral and Enteral Nutrition (ESPEN) recommends a protein intake of 1.0–1.5 g/kg/day in cancer patients [8]; additionally, oral supplementation with BCAAs (including leucine, isoleucine and valine) was proposed. Specifically, its use in combination with vitamin D resulted in improvements in muscle mass and lower-extremity function among older sarcopenic adults [9]; despite this, its use in combination with physical exercise failed to produce significant improvements in physical performance, nutritional status, fatigue or quality of life in patients with cancer [10].

In this context, the aim of this study is to compare the clinical evolution of patients with cancer when receiving oral nutritional supplementation with standard hypercaloric, hyperproteic (whey protein-based) OS versus hypercaloric, hyperproteic leucine-enriched oral supplements during a twelve-week period. Patients presented with primary tumors in different locations and were receiving systemic treatment (with chemotherapy, radiotherapy or a combination of both).

## 2. Material and Methods

### 2.1. Patients

This study was approved by the Ethics Committee of the Reina Sofia University Hospital (Cordoba, Spain; reference number 4788), which was conducted in accordance with the Declaration of Helsinki and according to national and international guidelines. This is a prospective open-label study, wherein a written informed consent form was signed by every individual before inclusion into the study. All patients received information before the inclusion and were only included if they agreed to participate. The inclusion criteria were the following: patients whose sex is male or female, aged > 18 years, with cancer of different origin treated with systemic treatment (chemotherapy, radiotherapy or combination treatment) that presented with weight loss > 5% during the previous three months or >10% during the previous six months. Exclusion criteria were the following: end-stage kidney disease and life expectancy < 2 weeks. Sample size was calculated based on the usual number of treated patients during 6 months in this hospital. Forty-six consecutive patients with cancer of different origin (Table 1) treated with systemic treatment chemotherapy, radiotherapy or combination treatment were included.

### 2.2. Study Design

**Figure 1:**
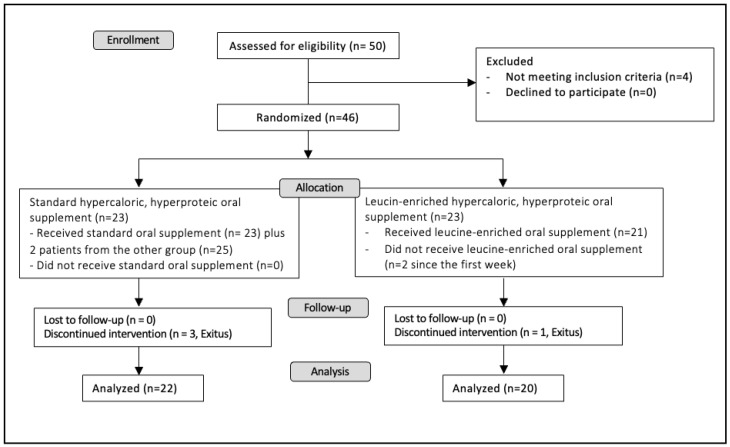
Study design.

When included in the study, all patients received general education about nutritional support, OS, Mediterranean diet and physical activity; additionally, patients received oral supplementation with calcifediol (in different doses to reach levels of sufficiency, defined with a serum 25OH vitamin D levels > 30 ng/dL) for avoiding confounding results. This was an open clinical trial. Patients were randomly assigned by the clinical investigator to receive either standard hypercaloric, hyperproteic (whey protein-based) OS or hypercaloric, hyperproteic, leucine-enriched oral supplement with a 1:1 allocation during a twelve-week period. Any orexigenic was used. A total of 46 patients were recruited, 23 patients for each arm; during the first week of the study, 2 patients reported nausea with the leucine-enriched oral supplement and switched arms. Nutritional evaluation was performed by the same endocrinologist in all cases; vectorial bioelectrical bioimpedance was performed using a NUTRILAB-Akern impedanciometer (Akern, Florence, Italy); for grip strength, a Jamar^®^ hydraulic dynamometer (Performance Health, Warrenville, IL, USA) was used and the nutritional echography was performed using a GE Logiq E9 ultrasound machine and a linear 9L-D probe (GE Healthcare, Chicago, IL, USA). Four patients died during the study period (three in the control arm group and one in the intervention arm group). A schematic overview of the design of this study is depicted in Figure 1. Both OS were hypercaloric, hyperproteic, with a comparable composition (details in Supplementary Table S1).

### 2.3. Outcomes

Primary outcome was to evaluate the percentage of change in muscle mass of patients after both nutritional interventions. Secondary outcomes included other changes in body composition (lean mass, water, bone, phase angle); anthropometric parameters (calf, arm and abdominal circumference); biochemical nutritional parameters (hemoglobin, lymphocytes, total cholesterol, total, high density lipoprotein (HDL) cholesterol, low density lipoprotein (LDL) cholesterol, triglycerides, transferrin, ferritin, albumin, prealbumin C-reactive protein (C-RP)); functionality (stand-up test and grip strength measured using Jamar^®^ hydraulic dynamometer); quality of life (measured using a self-rated health score through a 0–100 scale); ultrasound-measured nutritional variables including 1. abdominal fat mass ultrasound (total abdominal adipose tissue, subcutaneous adipose tissue, deep adipose tissue and pre-peritoneal fat) and 2. rectus femoris echography (adipose tissue, Y and X axis of the muscle, muscle area and circumference). This morphofunctional nutritional evaluation was performed as previously described [11].

### 2.4. Statistical Analysis

Between-group comparisons were analyzed using the Mann–Whitney U test (nonparametric data) or the Kruskal–Wallis test (nonparametric data, when we compared more than two groups). Paired analysis was performed using Student’s *t* test and Wilcoxon test (nonparametric data). Chi-square test was used to compare categorical data. Statistical analyses were performed using SPSS statistical software version 20, and Graph Pad Prism version 6. Data are expressed as mean ± SEM and percentages. The *p*-values < 0.05 were considered statistically significant.

## 3. Results

Forty-six patients were included. Most of them were female (54.3%), with a median age of 74 years; the patients presented with tumors of different origin, including colorectal cancer (19.6%), urothelial cancer (32.6%), head–neck cancer (13%), gastric cancer (8.7%), gastrointestinal neuroendocrine tumor (8.7%) and other localizations (17.4%). Over 63% of the evaluated patients underwent surgery, and almost 50% received combined treatment (Table 1). The clinical characteristics of both groups were similar, including gastrointestinal symptoms, weight loss, dependency level, self-rated health score and the Eastern Cooperative Oncology Group (ECOG) performance scale. Remarkably, a higher incidence of other neoplasms was observed in the intervention group compared with the control group (33.3% vs. 22.2%, *p* < 0.05); additionally, patients of the leucine-enriched group underwent radiotherapy more frequently (8% vs. 4%, *p* < 0.05). Any harm related with the study was referred by the participants.

### Primary and Secondary Outcomes

**Figure 2:**
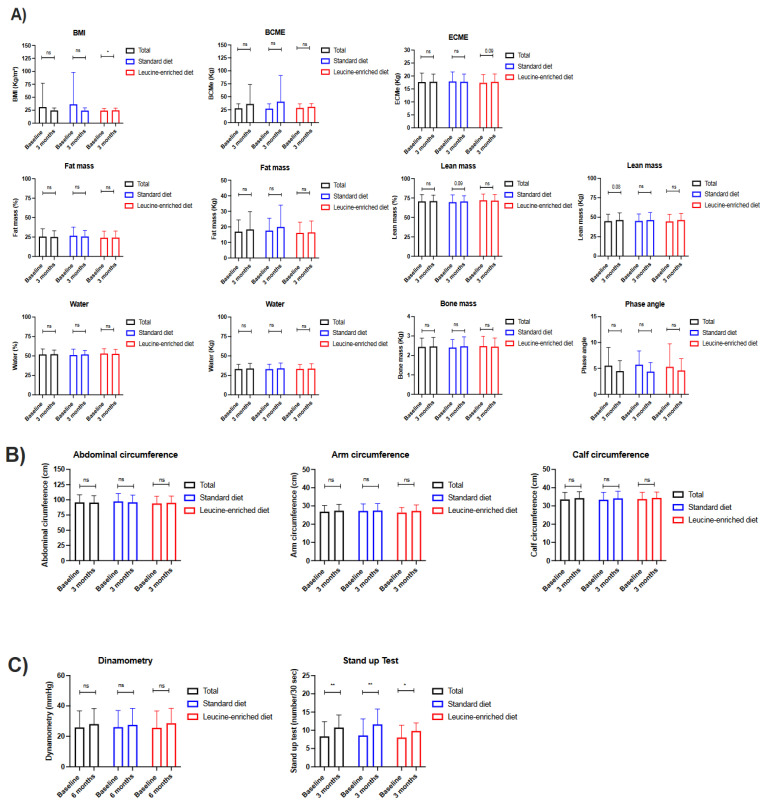
Clinical comparison in patients with cancer and systemic treatment receiving nutritional supplements with standard hypercaloric, hyperproteic oral supplements versus leucine-enriched oral supplements. (**A**) Bioimpedance analysis; (**B**) anthropometric analysis; (**C**) functional parameters. Legend: ns—non-significative; * *p* < 0.05; ** *p* < 0.01.

The nutritional intervention in all the evaluated patients resulted in body composition maintenance assessed via bioimpedance analysis. Particularly, the BMI decreased in a non-significant manner; furthermore, the lean mass tended to increase after twelve weeks of treatment (Supplementary Table S2; Figure 2A). When the control and intervention group are compared, the BMI increased 0.8 kg/m^2^ in the intervention group (*p* < 0.05), while it decreased in a non-significant manner in the control group. The extracellular mass (ECME) also increased in the intervention group (*p* < 0.05). Other parameters, including the fat, lean and bone mass, as well as the phase angle, remained stable after 12 weeks of intervention when both diets were compared (Figure 2A). Additional anthropometric parameters including abdominal, arm and calf circumference did not change after twelve weeks in the whole cohort, and when both groups are compared (Figure 2B).

Significantly, all patients improved their performance, which was measured using the stand-up test, especially those that received standard OS (control group); in contrast, the arm strength (determined via dynamometry) remained stable in all groups (Supplementary Tables S2 and S3; Figure 2C).

**Figure 3:**
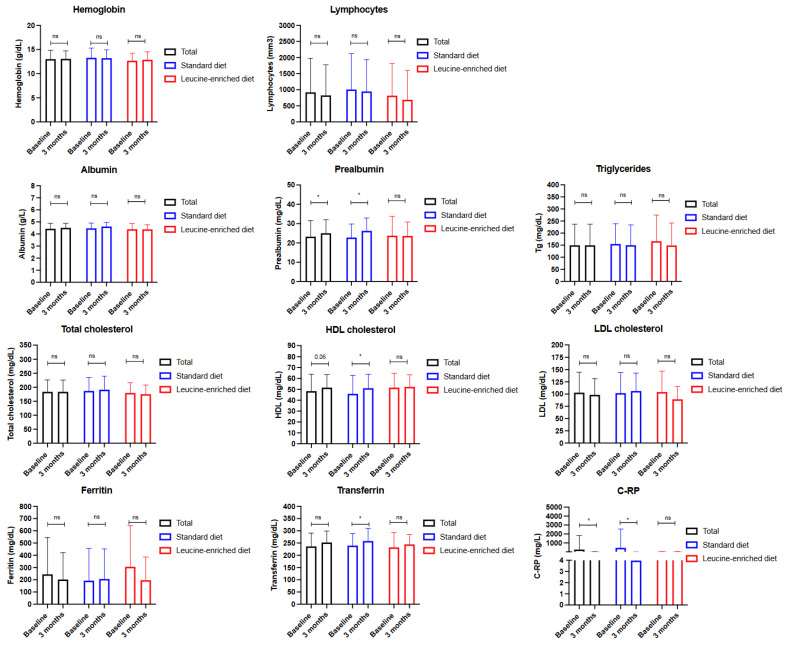
Biochemical comparison in patients with cancer and systemic treatment receiving nutritional supplements with standard hypercaloric, hyperproteic oral supplements versus leucine-enriched oral supplements. Legend: C-RP—c-reactive protein; ns—non-significative; * *p* < 0.05.

Regarding the biochemical data, when all the patients were analyzed, only the prealbumin levels increased (*p* < 0.05), and the C-reactive protein (C-RP) decreased (*p* < 0.05); additionally, the high-density lipoprotein cholesterol (HDL) levels tended to increase (*p* = 0.06; Figure 3; Supplementary Table S4). When the control and intervention groups were compared, the control group presented with significant increases in serum prealbumin, HDL cholesterol and transferrin levels; additionally, the C-RP also significantly decreased in this group (*p* < 0.05) compared with the intervention group (Figure 3). All of these changes are not only statistically significant, but also clinically relevant (Supplementary Table S5). Since all patients received specific oral supplements with calcifediol, the 25-OH vitamin D serum levels increased in all the patients, and all the patients reached sufficiency levels (25-OH vitamin D > 30 ng/dL; Supplementary Tables S4 and S5).

**Figure 4:**
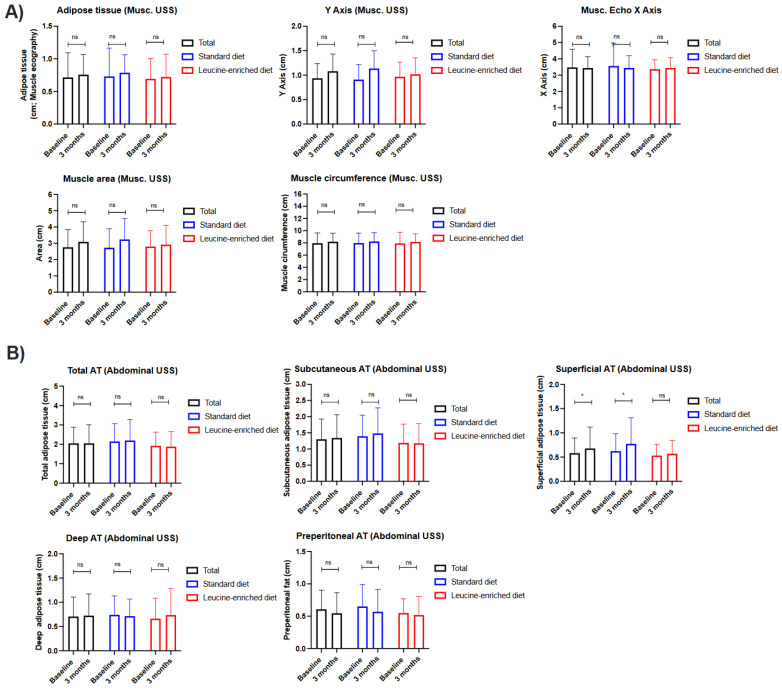
Clinical comparison in patients with cancer and systemic treatment receiving nutritional supplements with standard hypercaloric, hyperproteic oral supplements versus leucine-enriched oral supplements using ultrasound techniques. (**A**) Muscle echography of the rectus femoris muscle of the quadriceps; (**B**) abdominal adipose tissue ultrasound. Legend: ns—non-significative; * *p* < 0.05.

Finally, the nutritional evaluation performed using echography showed that the muscle mass in the rectus femoris of the quadriceps remained stable after twelve weeks of treatment in all the patients (Figure 4A). Additionally, the abdominal subcutaneous adipose tissue increased in the patients that received standard OS (Figure 4B; Supplementary Table S3).

## 4. Discussion

The nutritional status is severely affected in most patients with cancer; this condition affects the treatment tolerability and overall survival [5,12,13,14], and for these reasons, it is necessary to routinely screen for malnutrition and start early nutritional support [12,15]. Specifically, malnutrition affects 15–40% of cancer patients at diagnosis, and it is estimated that 40–80% of cancer patients will present malnutrition during the course of the oncological disease [16].

Malnutrition affects patients of both the male and female sexes and all age groups. In cancer, malnutrition is a result of a combination of anorexia, gastrointestinal symptoms and metabolic dysregulation, including insulin resistance [17]. It was suggested that malnutrition starts with anorexia due to an inflammatory state, and, consequently, weight and muscle loss are observed [18]. These changes are either caused by the tumor itself, or might be caused by treatment-related adverse effects [17]. Patients with or without malnutrition can present with sarcopenia, which was defined as an age-related, involuntary loss of skeletal muscle mass and strength; however, this muscle mass loss is not only observed in the elderly, but has also been related to disease. In this context, malnutrition is currently defined as a condition characterized by the loss of skeletal muscle mass and function. Recognizing sarcopenia, and not only malnutrition, is essential to select appropriate treatments and establish suitable recovery goals [19].

It is well known that nutritional support plays a fundamental role in the prevention, treatment and prognosis of both acute and chronic diseases, especially in cancer patients [20]. Despite this, clinical evidence in some scenarios is still lacking; most available clinical studies have a small sample size, where the nutritional intervention is short and, in most cases, is compared to placebo. Additionally, the included patients are usually affected by one or more systemic diseases, which adds confounding variables for appropriate analysis.

Currently, several specific nutritional formulas are available, and their use in precise clinical scenarios is recommended; for example, the use of immunonutrition as a part of abdominal surgery programs or even after surgery in patients with head–neck cancer is recommended [21,22]. Despite this, there is no specific recommendation for the use of specific OS in cancer patients undergoing systemic treatment. In this context, this study aims to compare the clinical effect of the nutritional support with standard hypercaloric, hyperproteic (whey protein-based) OS vs. hypercaloric, hyperproteic, leucine-enriched OS in patients with cancer undergoing systemic treatment (chemotherapy, radiotherapy or their combination).

In patients with cancer, an adequate supply of protein is necessary for the maintenance and gain of muscle. Specifically, positive muscle protein balance is necessary for increasing skeletal muscle mass; additionally, the availability of postprandial circulating amino acids is required for stimulating muscle protein synthesis [23]. Despite this, many patients with cancer do not meet this standard [24]. For this reason, current clinical guidelines recommend a high range of protein intake, 1.2–1.5 g/kg/day [5]; a review of cancer cachexia concluded that a dietary protein intake of >1.5 g/kg/day can maintain or improve muscle mass, and the intake of 2.0 g of protein/kg/day is associated with positive protein balance, especially when combined with exercise, even in patients with cachexia [25].

According to the American Cancer Society Nutrition and Physical Activity Guidelines for Cancer Survivors (ACS guidelines), a healthy body weight, physical activity and a diet rich in vegetables, fruits and whole grains was associated with a longer survival in stage III colon cancer patients. Additionally, they reported an inverse association between red meat consumption and seven-year mortality among 992 individuals with stage III colon cancer and suggested that a higher protein intake might be beneficial for these patients [25]. Unfortunately, most patients do not reach this goal with regular food intake, and nutritional supplementation with OS or protein powder is frequently required.

In all subjects, muscle protein synthesis and breakdown are simultaneous ongoing processes; thus, a positive balance should be presented in order to gain muscle mass. Several studies were focused on protein quality for muscle mass gain, since protein composition and amino acids can affect protein balance, and even exercise-induced muscle protein anabolism [26]. For these reasons, clinical studies using amino acids have a great interest in patients with cancer, since nutritional support aims to optimize nutritional status and counteract muscle mass wasting [18].

In this context, BCAAs (leucine, isoleucine and valine) have a critical role in promoting protein synthesis in muscle tissue, and for that reason, they were investigated as a target for nutritional therapy [24,27]. In contrast to our results, a previous study reported that a leucine-enriched OS induced a higher muscle protein fractional synthetic rate compared with a standard OS in patients with advanced cancer (25 patients with mixed tumor types during a four-week period) [28]. In this previous study, the composition of the leucine-enriched OS included 40 g of casein and whey-based protein, and it was additionally enriched with 4.16 g leucine, fish oil and oligo-saccharides; in contrast, the composition of the standard OS included 24 g of protein [28]. Differences observed between the previous study and our study might be explained by the significant difference in the protein content of both OS.

Vitamin D deficiency is frequent in cancers of different origin; its supplementation is strongly recommended for promoting bone health and reducing potential cancer risks [29]; additionally, several studies suggested an association between decreased vitamin D levels and increased mortality in cancer patients [30]. Several commercially available supplements are enriched with vitamin D in different proportions; for this reason, and in order to avoid confounding results, additional vitamin D supplementation with a similar biochemical goal (25OH vitamin D levels > 30 ng/dL) was offered to all patients. Additionally, previous studies reported that the use of vitamin D and leucine-enriched whey protein OS resulted in improvements in muscle mass and lower-extremity function among older adults with sarcopenia when compared to placebo [9]; additionally, when combined with exercise, it improved functionality, quality of life and even decreased inflammation [31], resulting in improved rehabilitation and early discharge [32]. Nutritional interventions using leucine-enriched OS also resulted in improvements in lower extremity function, and preserved muscle mass in patients with Parkinson’s disease or parkinsonism when compared with no intervention [33]. In our cohort, we did not observe increases in muscle mass, but it remained stable twelve weeks after the OS was started, suggesting a neutral muscle balance. The differences between our results and previously published studies might be explained by the fact that in our cohort, the patients had an underlying condition characterized by a catabolic state and chronic inflammation; additionally, they were receiving a systemic treatment, which may have limited the muscle mass gain. Despite this, improvements in physical performance and self-perceived quality of life were also observed in our study. Importantly, in this study, leucine-enriched OS was compared to a whey protein-based standard OS, and not with the placebo.

Regarding this, whey protein is a dairy-driven product that is usually used to help people change their weight and body composition and increase muscle strength. Specifically, whey protein is characterized by a significant content of essential and branched-chain amino acids; in consequence, it is a high-quality source of protein. It additionally has a rapid absorption and a higher muscle protein synthesis compared with other protein sources [34]. A recent metanalysis of thirty-five randomized clinical trials (1902 adult participants) showed that whey protein supplementation had a positive effect on lean body mass (0.741, 95% CI: 0.07, 1.41, *p* < 0.05) and a negative effect on other variables including BMI (−0.156, 95% CI: −0.31, 0.00, *p* < 0.05), body fat mass (−0.144, 95% CI: −0.28, 0.00, *p* < 0.05) and waist circumference (−0.448, 95% CI: −0.86, −0.03, *p* < 0.05) [35].

In contrast to the referred previous publications, a recent metanalysis demonstrated that leucine-isolated supplementation did not improve muscle mass and strength in elderly subjects. However, its combination with vitamin D exhibited a significant benefit for muscle strength, handgrip strength, gait speed and general performance in older adults [36]. During the past years, several publications highlighted the role of vitamin D in metabolism, as well as its role in frail patients, suggesting that its use might be related with reduced inflammation, improved insulin resistance and functionality (determined by decreased falls) [37,38,39].

Furthermore, some studies reported that leucine is linked to iron and hemoglobin metabolism [40]; moreover, its supplementation resulted in increased body weight and hemoglobin levels in patients with Diamond–Blackfan anemia [41]. We did not observe changes in the hemoglobin or ferritin levels; this finding might be explained by the fact that most included patients did not have anemia. Importantly, in these studies, leucine was offered as an oral complement and not as part of a nutritional OS. Remarkably, the increase in transferrin in the control group might be related to the composition of the standard OS, which is whey protein-based, and in consequence, it is rich in essential amino acids, which are also related to iron metabolism [41].

A recent review of pre-clinical studies found positive implications of leucine supplementation for reducing skeletal muscle loss (since leucin preserves protein synthesis and decreases protein degradation), attenuating cardiac dysfunction, improving immune competence, preserving energy production capacity and decreasing inflammation [4]. Furthermore, oral supplementation with BCAA was associated with a decreased incidence of hepatocellular carcinoma in patients with cirrhosis [42]; in contrast, other studies suggest that this supplementation may increase tumor growth [43]. Despite this, a recent review reports that there is insufficient evidence to establish a cause–effect relationship between leucine supplementation and tumor growth [4]. All these mechanisms require further investigation since the current available studies that specifically evaluate the use of leucine-rich feeding during cancer cachexia is limited to pre-clinical rodent studies.

Previous publications suggested a specific effect of leucine on decreasing inflammation [31]; furthermore, in vivo studies suggested that leucine-enriched amino acids accelerate recovery from muscle damage by preventing excessive inflammation in a rat model [44]. When the C-RP values were analyzed, we did not observe this effect in the intervention group, but inflammation diminished in the control group. This situation is probably explained by the fact that more patients in the intervention group received radiotherapy compared with the control group, which is associated with increased tissue inflammation [45,46].

This study has some limitations, with the first limitation being the number of participants in each group and the heterogeneity of the primary tumor location. This was also a short intervention (12 weeks), and the daily intake of OS (using an adherence scale) was not assessed. Additionally, tumor markers were not included. Several reasons explain this exclusion; first, tumor progression was not the goal of this study, and the primary site and stage at the diagnosis were heterogenous, which would have limited the conclusions. It is important to mention that the treatment tolerance was similar in both groups after the first week and until the twelfth week of treatment, but specific day-per-day adherence was not assessed in this study. The gastrointestinal symptoms are frequent in patients with cancer due to systemic treatment-related adverse effects and the local effects of the tumor [5], which might influence the complete adherence to the nutritional support with both OS, and might mask some effects of the leucine-enriched formula.

In contrast, this study has several strengths; first of all, it is a real reflection of the clinical practice, in which patients with tumors of different locations and stages undergo different lines of systemic treatment and require an appropriate nutritional evaluation combined with early nutritional support. Additionally, we performed a head-to-head comparison of two different OS in cancer patients undergoing systemic treatment (we did not use the placebo in the control group). Furthermore, a comprehensive nutritional evaluation was performed, including anthropometric, echography, functional and biochemical parameters; finally, vitamin D supplementation was included in order to avoid confounding results.

## 5. Conclusions

Taken together, our results reveal that the use of nutritional interventions using hypercaloric, hyperproteic OS results in the stabilization of the nutritional status and improvements in physical performance and quality of life in patients with cancer undergoing systemic treatment with chemotherapy, radiotherapy or their combination. These effects were independent of the vitamin D supplementation. We did not observe significant differences when the hypercaloric, hyperproteic, whey protein-based standard OS was compared with the hypercaloric, hyperproteic, leucine-enriched OS. Additional head-to-head studies comparing different OS formulas should be performed in patients with cancer, especially in larger and more homogenous cohorts; furthermore, specific studies for evaluating the role of leucine-enriched OS in tumor progression should be designed.

## Figures and Tables

**Table 1:** Baseline clinical characteristics of the patients. Comparison between groups based on the nutritional intervention.

Characteristics	Total (*n* = 46)	Standard OS (*n* = 25)	Leucine-Enriched OS (*n* = 21)	*p*
Sex (♂/♀)	45.7/54.3% (21/25)	48/52% (12/13)	42.9/57.1% (9/12)	0.48
Age at diagnosis (years)	74.5 (71–78)	66 (56–74)	71 (53–78)	0.7
Tobacco exposure				0.26
No.	50% (21/42)	54.2% (13/24)	44.4% (8/18)	
Active	28.6.% (12/42)	33.3% (8/24)	22.2% (4/18)	
Previous exposure	21.4% (9/42)	12.5% (3/24)	33.3% (6/18)	
Previous other neoplasms	19.7% (9/46)	8% (2/25)	33.3% (7/21)	0.04
Tumor localization				
Head/neck	13% (6/46)	12 (3/25)	14.3% (3/21)	
Gastrointestinal NET	8.7% (4/46)	16 (4/25)	0%	
Gastric cancer	8.7% (4/46)	8 (2/25)	9.5 (2/21)	
Colorectal cancer	19.6% (9/46)	12 (3/25)	28.6% (6/21)	
Urothelial	32.6% (15/46)	24 (7/25)	9.5% (2/21)	
Other	17.4.% (8/46)	28 (6/25)	38.1 (8/21)	
Treatment				
Surgery	63% (29/46)	68% (17/25)	55.1% (12/21)	0.33
Chemotherapy	56.5% (26/46)	52% (13/25)	61.9% (13/21)	0.35
Radiotherapy	19.6% (9/46)	4% (1/25)	8% (8/21)	0.005
Combined treatment	47.8 (22/46)	48% (12/25)	47.6% (10/21)	0.61
Symptoms				
Weight loss (3 months)	84.4% (38/45)	79.2% (19/24)	90.5% (19/21)	0.27
Weight loss in kg (3 months)	5 (4–6)	3 (0–19)	6 (2–6)	0.5
Weight loss (6 months)	71.7% (33/46)	60% (15/25)	85.7% (18/21)	0.053
Weight loss in kg (6 months)	6.5 (6–7)	3 (0–19)	6 (4–10)	0.8
Gastrointestinal symptoms	43.5% (20/46)	36% (9/25)	52.4% (11/21)	0.21
Abdominal pain	32.6% (15/46)	24% (6/25)	42.9% (9/21)	0.15
Nausea/vomiting	22.2% (10/45)	16.7% (4/25)	28.6% (6/21)	0.27
Diarrhea	15.2% (7/46)	16% (4/25)	14.3 (3/21)	0.60
Dyspnea	17.4% (8/46)	20% (5/25)	14.3% (3/21)	0.46
Mucositis	8.7% (4/46)	4% (1/25)	14.3% (3/25)	0.24
Quality of life				
Any level of dependency	43.5% (20/46)	44 (11/25)	42.9 (9/21)	0.59
Self-rated health score	65 (0–80)	70 (35–84)	60 (45–75)	0.3
ECOG				0.3
ECOG 0	60.9 (28/46)	60 (15/25)	61.9 (13/21)	
ECOG 1	28.3 (13/46)	28 (7/21)	28.6 (6/21)	
ECOG 2	10.9 (5/46)	12 (3/25)	9.5 (2/21)	
Mortality	8.7 (4/46)	12 (3/25)	4.8% (1/25)	0.37

Legend: NET: neuroendocrine tumor; ECOG: ECOG performance status scale; RT: radiotherapy.

## Data Availability

All data was included in the article.

## Supplementary Materials

The following supporting information can be downloaded at: https://www.mdpi.com/article/10.3390/nu15122726/s1.
