# Supplementary material for: Standard Hypercaloric, Hyperproteic vs. Leucine-Enriched Oral Supplements in Patients with Cancer-Induced Sarcopenia, a Randomized Clinical Trial
Source: Nutrients. 2023 Jun 12;15(12):2726. doi: 10.3390/nu15122726 (PMC10302788; doi:10.3390/nu15122726)
Supplement: Supplementary file 1 [file nutrients-15-02726-s001.zip › nutrients-2432680-supplementary.pdf]

Table S1. Composition for 100 ml of the standard and leucine-enriched oral supplements

Characteristics	Standard	Leucine-enriched
**Kcal**	160	151
**Energeric density (Kcal/mL)**	1.6	1.51
**Fat** (g)	6.7	5.2
Saturated (g)	0.8	0.46
Monounsaturated (g)	3.9	
Polyunsaturated (g)	1.8	
**Carbohydrates** (g)	15.7	14,8
Sugar (g)	9.5	8,1
Lactose (g)	0.9	< 0.2
**Proteins** (g)	9.8	10.4
Serumproteins (g)	1.4	9.7
Casein (g)	8.4	0
L-leucin (g)	0	1.5
**Vitamin D (mcg)**	1.2	5
**Osmolality** (mOsm/L)	540	680

Table S2. Morphofunctional assessment of the nutritional status at baseline and three months after nutritional support in all patients

Characteristics	Baseline (n=46)	Three months (n=42)	*p1*
**Body weight**	63.6 (40-89)	60.3 (42-92)	0.2
**Bioimpedance analysis**			
BMI (Kg/m2)	23.6 (16.6-33)	23.8 (16.1-32.9)	0.5
BCMe	30 (17.9-41.3)	29.6 (10.2-41.8)	0.2
ECMe	17.6 (11.3-22.8)	18 (12.3-23.4)	0.2
Fat mass (%)	27 (12.6-59.3)	26.8 (8.7-40.7)	0.2
Fat mass (Kg)	17 (7-33.2)	16.4 (4.2-33.7)	0.6
Lean mass (%)	69.2 (56.9-83.1)	69.6 (56-86.7)	0.2
Lean mass (Kg)	45.4 (25.2-60.9)	45.6 (29.8-62)	0.08
Water (%)	50.2 (42.8-64.2)	51.7 (42.3-63.9)	0.2
Water (Kg)	32.5 (20.8-44.2)	33.5 (21.9-46.2)	0.1
Bone Mass (Kg)	2.4 (1.5-3.2)	2.4 (1.6-3.2)	0.6
**Phase angle**	4.5 (3.6-5.9)	4 (3.5-5.6)	0.12
**Anthropometric evaluation**			
Abdominal circumference	95 (76-126)	91 (76-117)	0.2
Arm circumference	26 (21-33)	27 (20-34)	0.2
Calf circumference	33 (26-40)	34 (27-40)	0.9
**Muscle Ecography**			
Adipose tissue	0.64 (0.21-2.31)	0.74 (0.34-1.44)	1
Area	2.5 (0.96-5)	3 (1-5.7)	0.6
Circunference	7.7 (2.5-11)	8.3 (5.6-10.7)	0.9
AP axis	0.9 (0.36-1.48)	0.98 (0.56-1.77)	0.9
Transversal axis	3.24 (2.39-9.34)	3.58 (1.8-4.2)	0.2
**Abdominal ecography**			
Total adipose tissue	1.8 (1-4)	1.9 (0.8-4.8)	0.8
Subcutaneous adipose tissue	1.15 (0.9-1.51)	1.16 (0.8-1.7)	0.9
**Superficial subcutaneous adipose tissue**	**0.51 (0.37-0.68)**	**0.59 (0.36-0.8)**	**0.02**

Deep subcutaneous adipose tissue	0.6 (0.1-2.5)	0.59 (0.1-2.3)	0.7
Preperitoneal adipose tissue	0.5 (0.3-1.3)	0.5 (0.08-1.4)	0.3
**Functional evaluation**			
Dynamometry (dominant arm)	26 (7-51)	26 (14-50)	0.1
**Stand up test**	**9 (0-17)**	**10 (4-22)**	**0.03**

p refers to the comparison between patients at baseline and after three months of nutritional support with OS

Table S3. Morphofunctional assessment of the nutritional status at baseline and three months after nutritional support with standard and leucine-enriched OS

	Standard OS			Leucine-enriched OS		
**Characteristics**	**Baseline (n=25)**	**Three months (n=22)**	***p1***	**Baseline (n=21)**	**Three months (n=20)**	***p2***
**Body weight**	65.8 (51-89)	60.6 (42-92)	0.9	**60 (39-80)**	**60.3 (44-92)**	**0.03**
**Bioimpedance analysis**						
BMI (Kg/m2)	24 (19.6-30.2)	21.5 (16.6-29)	0.5	**23.4 (16.6-29)**	**24.2 (16.1-31.5)**	**0.03**
BCMe	29.9 (22.6-41.3)	28.2 (10.2-39.5)	0.3	27.8 (21.5-38.4)	30.8 (21.4-41.8)	0.3
ECMe	17.6 (13.7-22.8)	17.9 (12.3-23.4)	0.8	15.5 (11.3-21.5)	18.5 (12.9-23.4)	0.09
Fat mass (%)	27.4 (17.7-36.5)	26 (9.9-40.7)	0.2	23.9 (16.1-34.6)	26 (8.7-37.4)	0.9
Fat mass (Kg)	14.7 (10.8-27.7)	15.5 (4.2-33.7)	0.5	16.1 (7-2.7)	16.4 (5-31.6)	0.8
Lean mass (%)	68.9 (60.3-78.2)	69.5 (56-85)	0.09	71.9 (62-79.7)	70 (59.5-86.7)	0.9
Lean mass (Kg)	45 (34.8-60.9)	45.4 (29.8-60.4)	0.5	41.1 (25.2-56.9)	46.8 (32.5-62)	0.1
Water (%)	50.2 (44.7-58.8)	51.8 (42.3-63.1)	0.1	53.4 (45.7-64.2)	51.7 (44.4-63.9)	1
Water (Kg)	32.2 (25.8-44.2)	32.8 (21.9-46.2)	0.4	30.7 (24.7-42)	34.2 (24.2-45.7)	0.3
Bone Mass (Kg)	2.4 (1.9-3.2)	2.4 (1.6-3.2)	0.3	2.2 (1.7-3)	2.5 (1.8-3.2)	0.1
Phase angle	5.7 (3.3-13.7)	4.3 (2.1-6.8)	0.16	5.3 (3.3-22.1)	4.6 (2.7-6.7)	0.44
**Anthropometric evaluation**						
Abdominal circumference	98 (76-113)	90 (76-117)	0.3	90 (72-107)	93 (77-113)	0.3
Arm circumference	26 (24-32)	27 (20-34)	0.3	26 (21-31)	26 (21-34)	0.1
Calf circumference	33 (26-39)	33 (27-39)	0.9	33 (27-38)	34 (28-40)	0.9
**Muscle Ecography**						
Adipose tissue	0.69 (0.38-0.97)	0.85 (0.53-1.37)	0.4	0.53 (0.31-1.21)	0.7 (0.34-1.44)	0.2
Area	3 (1.7-4.2)	3 (1.6-5.7)	0.2	2.6 (1-5)	2.9 (1-5.3)	0.6
Circunference	8.4 (6.3-9.5)	8.3 (5.6-10.7)	1	8.1 (5.7-9.6)	8.3 (5.7-10.8)	0.9
AP axis	1.13 (0.46-1.44)	1.02 (0.72-1.77)	0.1	0.86 (0.36-1.48)	0.82 (0.56-1.68)	0.8
Transversal axis	3.28 (2.58-9.34)	3.53 (1.8-4.2)	0.8	3.48 (2.6-4.02)	3.7 (2.3-4.2)	0.9
**Abdominal ecography**						
Total adipose tissue	1.9 (1-3)	1.9 (0.8-4.8)	0.7	1.5 (1-2.6)	1.8 (1-4)	0.5

Subcutaneous adipose tissue	1.2 (0.4-2.25)	1.3 (0.4-3.3)	0.7	1 (0.4-1.9)	1 (0.5-2.9)	0.5
Superficial subcutaneous adipose tissue	**0.56 (0.42-0.71)**	**0.64 (0.46-0.82)**	**0.03**	0.47 (0.32-0.66)	0.55 (0.31-0.70)	0.4
Deep subcutaneous adipose tissue	0.6 (0.2-1.5)	0.69 (0.1-1.4)	0.6	0.5 (0.1-1)	0.6 (0.2-2.3)	0.9
Preperitoneal adipose tissue	0.5 (0.3-1)	0.5 (0.08-1.3)	0.3	0.5 (0.3-0-8)	0.4 (0.3-1.4)	0.6
**Functional evaluation**						
Dynamometry (dominant arm)	27.3 (7-51)	25 (14-47)	0.2	24 (8-40)	28 (17-51)	0.5
Stand up test	**9.5 (5-11)**	**11 (6-10)**	**0.008**	**8 (6-10)**	**9.8 (8-12)**	**0.02**

*p*1 refers to the comparison between patients that received standard OS at baseline and after three months; *p*2 refers to the comparison between patients that received leucine-enriched OS at baseline and after three months

Table S4. Biochemical analysis at baseline and three months after nutritional support in the whole cohort

Characteristics	Baseline (n=46)	Three months (n=42)	*p1*
**Biochemical parameters**			
Haemoglobin	13.5 (8.7-16.2)	13.2 (9.6-16)	0.7
Lymphocytes	990 (600-3590)	2720 (600-2900)	0.7
Albumin (g/dl)	4.5 (3.8-5.3)	4.5 (3.8-5.2)	0.5
Prealbumin (mg/dl)	**23 (8.9-56)**	**24 (5-36)**	**0.02**
Ferritin (mg/dl)	152 (5-1213)	155 (3-974)	0.6
Transferrin (mg/dl)	235 (123-342)	256 (133-347)	0.2
Total cholesterol (mg/dl)	182 (100-281)	176 (106-261)	0.6
HDL cholesterol	47 (29-81)	52 (29-72)	0.06
LDL cholesterol	106 (51-195)	99 (44-164)	0.8
Triglycerides	134 (73-468)	130 (87-393)	0.7
RCP	**5.1 (0.5-10309)**	**2.8 (0-147)**	**0.03**
Vitamin D	**12 (7-30)**	**33 (16-68)**	**<0.001**

p refers to the comparison between patients at baseline and after three months of nutritional support with OS

Table S5. Biochemical analysis at baseline and three months after nutritional support with standard and leucine-enriched OS

	Standard OS			Leucine-enriched OS		
**Characteristics**	**Baseline (n=25)**	**Three months (n=22)**	***p1***	**Baseline (n=21)**	**Three months (n=20)**	***p2***
**Biochemical parameters**						
Haemoglobin	14.3 (8.7-15.4)	13.4 (9.9-15.9)	0.6	13 (12.1-16.2)	13.1 (9.6-16)	0.9
Lymphocytes	1800 (1020-3590)	1120 (920-2900)	0.7	2100 (600-2340)	2600 (600-1800)	0.2
Albumin (g/dl)	4.4 (3.8-5.2)	4.7 (3.8-5)	0.2	4.9 (4-5.3)	4.4 (3.8-5.2)	0.7
Prealbumin (mg/dl)	**19.4 (8.9-30.9)**	**23 (14-36)**	**0.004**	25.9 (13.9-56)	24 (5-33)	0.2
Ferritin (mg/dl)	138.8 (52.3-1213)	128 (3-974)	0.8	173 (15-768)	144 (68-279)	0.4
Transferrin (mg/dl)	**219 (158-305)**	**279 (113-296)**	**0.05**	235 (188-337)	248 (133-296)	0.8
Total cholesterol (mg/dl)	153 (100-277)	197 (106-261)	0.9	174 (123-227)	173 (125-239)	0.5
HDL cholesterol	**41 (29-74)**	**50 (30-71)**	**0.03**	53 (44-81)	53 (29-72)	0.6
LDL cholesterol	93 (51-187)	108 (51-164)	0.5	94 (46-177)	96 (44-152)	0.7
Triglycerides	131 (73-181)	110 (89-393)	0.7	114 (93-468)	135 (87-231)	0.4
RCP	**8.6 (0.5-10309)**	**1.7 (0-17)**	**0.03**	1 (0-47)	3.6 (0-147)	0.4
Vitamin D	**15.4 (8-24)**	**34 (22-66)**	**<0.001**	**11 (8-33)**	**32 (16-68)**	**0.01**

*p*1 refers to the comparison between patients that received standard OS at baseline and after three months; *p*2 refers to the comparison between patients that received leucine-enriched OS at baseline and after three months
